# Family Physicians’ Knowledge Levels and Awareness Regarding HPV Infection and HPV Vaccines

**DOI:** 10.3390/healthcare14162457

**Published:** 2026-08-09

**Authors:** Fatma Ebru Yurdakul, Ömer Acat, Latife Uzun

**Affiliations:** 1 Department of Family Medicine, Faculty of Medicine, Karamanoğlu Mehmetbey University, 70200 Karaman, Türkiye; 2 Department of Public Health, Faculty of Medicine, Karamanoğlu Mehmetbey University, 70200 Karaman, Türkiye; omeracat@kmu.edu.tr; 3 Karaman Central No. 5 Family Health Center, 70100 Karaman, Türkiye; uzunlatife90@gmail.com

**Keywords:** cervical cancer, human papilloma virus, HPV vaccine, family physician, awareness, Türkiye

## Abstract

**Background**: In primary care settings, cervical cancer screening and prevention are highly dependent on healthcare provider initiatives, particularly in countries like Türkiye where the HPV vaccine is not yet integrated into the national routine immunization schedule. Since HPV vaccines provide over 90% protection against cervical cancer, the proactive guidance of primary care providers is critical. This study evaluated family physicians’ knowledge levels and awareness of HPV infection and human papillomavirus (HPV) vaccines in primary care settings in Türkiye. **Methods**: A total of 238 family physicians working in Türkiye participated in this descriptive cross-sectional study using a convenience sampling method. Data were collected through an online questionnaire administered via Google Forms. The survey included a sociodemographic information form and a 33-item HPV knowledge scale. Data were analyzed using descriptive statistics, normality assessment, chi-square test, Mann–Whitney U test, Kruskal–Wallis test, and hierarchical logistic regression analysis. **Results**: Overall, 81.1% of participants recommended the HPV vaccine, and 83.6% believed it should be included in the national schedule. Notably, only 15.1% of the participating physicians had received the HPV vaccine themselves, whereas 40.6% of the unvaccinated subgroup (82/202) stated they were considering personal vaccination. HPV knowledge levels were generally high, but lower when regarding the current national program. Vaccine recommenders and female participants had significantly higher knowledge scores, while out-of-pocket vaccine cost emerged as a major barrier to clinical recommendation. In multivariable hierarchical logistic regression, individual knowledge domains did not emerge as independent standalone predictors after controlling for demographics, with only knowledge of the current national program showing a borderline trend (*p* = 0.066), although the inclusion of the knowledge variables as a collective block significantly improved the overall model explanatory power (*p* = 0.003). **Conclusions**: Family physicians in Türkiye showed generally high HPV-related knowledge and a high rate of HPV vaccine recommendation. This study clarifies that primary care advocacy is shaped by the combined influence of professional knowledge and overriding structural factors, rather than individual knowledge domains acting as independent standalone predictors. While future strategies must focus on developing targeted continuous medical education to bridge specific knowledge gaps and optimize opportunistic counseling, achieving optimal immunization coverage ultimately relies on integrating the vaccine into national programs to mitigate out-of-pocket cost barriers.

## 1. Introduction

Human papillomavirus (HPV) is one of the most common sexually transmitted infections and is associated with substantial morbidity and mortality due to its role in the development of cervical, anal, vaginal, vulvar, penile, and oropharyngeal cancers, as well as genital warts [1,2,3]. More than 100 HPV genotypes have been identified, and at least 15 of these are considered high-risk types for cervical cancer. Among them, HPV-16 and HPV-18 are the most prevalent oncogenic genotypes and account for approximately 70% of cervical cancer cases worldwide [4,5]. An additional 20% of cases are attributed to five other high-risk genotypes, namely HPV-31, HPV-33, HPV-45, HPV-52, and HPV-58 [6].

Cervical cancer is largely preventable and, when detected early, can be successfully treated [7]. Nevertheless, it remains the fourth-most common cancer among women worldwide, accounting for 6.9% of all female cancers [8]. In 2020, nearly 90% of cervical cancer-related deaths occurred in low- and middle-income countries [9,10,11]. In Türkiye, cervical cancer ranks among the ten most common cancers in women and is reported as the eighth-most frequent type, with a proportion of 3.1% and an incidence of 4.7 per 100,000 women [12].

Following the recognition of HPV as the principal etiological factor for cervical cancer, HPV vaccination has become a cornerstone of primary prevention strategies, particularly among preadolescent girls and young women [4]. In addition to vaccination, regular cervical cancer screening in accordance with national recommendations remains essential to achieve early detection [1]. In this context, the World Health Organization (WHO) has set the 2030 targets of vaccinating 90% of girls by the age of 15 years, screening 70% of women at 35 and 45 years of age using a high-performance test, and ensuring treatment for 90% of women with cervical precancerous lesions or cervical cancer [1].

However, HPV-related disease is not limited to women. Previous studies have shown that HPV prevalence is also high among males older than 15 years, with approximately one in five men worldwide infected with at least one HPV type. Sexually active males, regardless of age, constitute an important reservoir for genital HPV infection. Reflecting this evidence, major international immunization guidelines—such as those in the United States—have long established gender-neutral vaccination schedules that routinely include both girls and boys [13,14].

As of late February 2025, 148 WHO member states had incorporated the HPV vaccine into their national immunization programs [15]. In addition, several Latin American countries, including Brazil, Costa Rica, the Dominican Republic, and Mexico, have expanded their HPV vaccination programs to include boys [16]. Currently, three licensed prophylactic HPV vaccines are available: the quadrivalent vaccine Gardasil^®^, which protects against HPV-6, HPV-11, HPV-16, and HPV-18; the bivalent vaccine Cervarix^®^, targeting HPV-16 and HPV-18; and the nonavalent vaccine Gardasil 9^®^, which additionally covers HPV-31, HPV-33, HPV-45, HPV-52, and HPV-58 [17]. In Türkiye, bivalent and quadrivalent HPV vaccines have been available and clinically recommended since 2007. Although official initiatives were launched in 2022 to evaluate the integration of the vaccine into the national routine immunization program, it has not yet been included in the free public vaccination schedule [18]. This ongoing exclusion from the national program is primarily driven by substantial economic costs and the fact that the vaccine remains a fully imported pharmaceutical product, which means that it is still accessible only on a voluntary, out-of-pocket basis for the general population. This structural framework highlights the distinct and complementary pathways of cervical cancer prevention within the Turkish primary healthcare system. While the Ministry of Health provides comprehensive, fully subsidized population-based screening initiatives (utilizing highly effective co-testing with HPV-DNA and Pap smears) through Family Health Centers and Cancer Early Diagnosis, Screening, and Training Centers (KETEM), HPV vaccination is currently available on a voluntary, out-of-pocket basis. Within this specific setting, the proactive clinical counseling, guidance, and recommendation behavior of family physicians serves as a vital bridge. As trusted primary care providers, their educational role is instrumental in aligning primary prevention with existing screening infrastructures, helping patients navigate preventive health choices, and addressing vaccine hesitancy.

Evidence from clinical and population-based studies indicates that HPV vaccines provide over 90% protection against cervical cancer [19,20,21]. Accordingly, improving HPV vaccine uptake is a key component of global efforts to eliminate cervical cancer as a public health problem. In primary care, family physicians play a central role in this process through their knowledge of HPV infection, HPV screening, and HPV vaccination, as well as their vaccine recommendation practices. The international literature strongly emphasizes the pivotal role of healthcare providers in immunization success, demonstrating that a direct provider recommendation is a key determinant of vaccine uptake [22,23]. For instance, a comprehensive systematic review involving 265,083 patients confirmed a significant association between clinician advocacy and the initiation of the HPV vaccination series [24]. Despite their clinical importance, several global barriers constrain provider recommendations. Key impediments identified across multi-country systematic reviews and qualitative guidelines include persistent doubts regarding vaccine safety, concerns over potential side effects, and diminishing trust in health authorities [23,25]. Knowledge gaps or misconceptions often lead to hesitant or omitted clinical recommendations, directly fueling public vaccine hesitancy and lowering immunization coverage [26,27]. Beyond professional knowledge, behavioral frameworks demonstrate that a clinician’s personal background, particularly parental status and the gender of their children, can operate as crucial cognitive determinants of preventive care advocacy. Experiential crossover literature shows that healthcare providers who are parents—especially those raising daughters—frequently exhibit greater clinical empathy and a heightened personal awareness of reproductive health risks, which directly translates into more proactive vaccine counseling and a stronger propensity to recommend the HPV vaccine in routine practice [28,29]. Consequently, evaluating the baseline knowledge of primary care physicians is a critical prerequisite for identifying communication barriers and designing targeted public health interventions. Therefore, this study aimed to assess the knowledge levels and awareness of family physicians working in Türkiye regarding HPV infection and HPV vaccination. Specifically, this study sought to address key secondary research sub-questions to provide a comprehensive, multi-dimensional analysis of primary care predictors: do these HPV-related knowledge domains and subsequent clinical recommendation practices vary significantly by the physicians’ gender and age, and to what extent are they influenced by distinct professional and personal backgrounds, including professional group (non-specialist family physicians, specialists, and residents), personal HPV vaccination history, parental status, and crucially, their own children’s gender and specific immunization status?

## 2. Materials and Methods

### 2.1. Study Design

The study population comprised physicians actively practicing within the primary care family medicine system in Türkiye. To ensure strict international terminological and methodological clarity, the overarching term ‘family physicians’ is used throughout this manuscript to describe all primary care providers within this framework, which structurally encompasses three distinct professional subgroups based on their training status: (1) non-specialist Family Physicians, referring to primary care medical doctors without formal post-graduate residency training who have completed the mandatory national family medicine orientation training program authorized by the Ministry of Health; (2) family Medicine Specialists, who have successfully completed the formal three-year post-graduate residency training program and obtained official board certification; and (3) family Medicine Residents, who are currently undergoing their active, formal post-graduate residency training in family medicine. All data were collected and processed in accordance with the principles of confidentiality and data protection. While a centralized institutional registry of all active family physicians exists under the Turkish Ministry of Health, individual contact rosters are strictly confidential and legally inaccessible for independent academic surveys due to national Personal Data Protection Laws (KVKK). Consequently, to ensure legal compliance and absolute participant anonymity, a non-probability convenience sampling approach was utilized. Participants were recruited through a digital invitation model, where the survey link was distributed across various professional primary care networks and medical messaging groups. This cross-sectional study was reported in accordance with the Strengthening the Reporting of Observational Studies in Epidemiology (STROBE) statement guidelines for cross-sectional studies (Supplementary File S1).

### 2.2. Inclusion and Exclusion Criteria

Participants were eligible for inclusion if they were actively working in Türkiye as a non-specialist family physician, family medicine specialist, or family medicine resident, agreed to participate in the study, and completed the questionnaire. Individuals who were not working in these roles, did not complete the questionnaire, or declined to participate were excluded from the study.

### 2.3. Study Instrument

This study was conducted as an online survey using Google Forms (Google LLC, Mountain View, CA, USA) designed to assess knowledge and awareness of HPV infection and HPV vaccination. The survey was distributed online to non-specialist family physicians, family medicine specialists, and family medicine residents working in Türkiye. The minimum a priori sample size threshold was calculated as 163 participants, based on an exploratory 80% confidence level and a 5% margin of error to account for anticipated online recruitment constraints; however, data collection was extended to achieve a final sample of 238 participants, successfully satisfying conventional statistical precision parameters. Data collection was carried out between July and December 2024, and a total of 238 participants voluntarily participated in the study. Recruitment was operationalized by disseminating the web-based survey link through major national primary care networks, closed medical forums, and secure instant messaging communication channels managed by prominent Turkish family medicine organizations and professional federations. Because the survey link was distributed across these broad digital physician networks rather than via direct personalized invitations, the total number of individual clinicians exposed to the link could not be logged as a static denominator. As a result, an exact mathematical response rate cannot be derived, which represents a standard procedural attribute of open-link online surveys. Nonetheless, data collection was maintained until the final pool of valid, fully completed submissions (*n* = 238) successfully surpassed our predefined sample size requirements.

The questionnaire consisted of two sections. The first section was developed by the researchers based on a comprehensive review of the relevant literature [22,23,24,25] and included 12 items assessing sociodemographic and professional characteristics, such as age, marital status, professional status, and HPV vaccination and recommendation status. Specifically, the primary outcome of clinical vaccine recommendation behavior was evaluated using a direct, binary question: ‘Do you recommend the HPV vaccine to your patients in your routine clinical practice?’ (coded as Yes = 1/No = 0). This item was specifically designed to capture the active integration of vaccine counseling into the physicians’ current primary care practice, rather than capturing historical single-instance recommendations (‘ever recommended’) or restrictive compliance thresholds. The second section included the HPV Knowledge Scale, originally developed by Waller et al. in 2013 [30] and later adapted, validated, and tested for reliability in Türkiye by Bozkurt and Özdemir (2023) [31]. The Turkish version of the scale consists of 33 items and four domains: general HPV knowledge, knowledge of HPV screening tests, knowledge of HPV vaccine protection, and knowledge of the current HPV vaccination program. Each correct response was scored as 1, whereas incorrect or “I do not know” responses were scored as 0. Total scores ranged from 0 to 33, with higher scores indicating greater HPV-related knowledge. To ensure conceptual and methodological clarity, ‘knowledge’ and ‘awareness’ were operationalized as distinct dimensions within our study design. While ‘knowledge’ was strictly evaluated as an objective metric using the total score derived from the validated clinical HPV knowledge scale, ‘awareness’ was operationalized as the physicians’ baseline familiarity with and systemic recognition of national public health frameworks. Specifically, awareness was measured through categorical and institutional items evaluating the participants’ recognition of the current national routine immunization schedule, updates regarding national screening guidelines, and the regulatory status of HPV vaccines in Türkiye.

### 2.4. Statistical Analysis

All statistical analyses were performed using IBM SPSS Statistics for Windows, version 25.0 (IBM Corp., Armonk, NY, USA). Categorical variables were summarized as frequencies and percentages, whereas continuous variables were presented as mean ± standard deviation or median (interquartile range, 25th–75th percentile), as appropriate according to data distribution. Because the scale items were dichotomously scored as correct = 1 and incorrect or “I do not know” = 0, internal consistency was assessed using the Kuder–Richardson Formula 20 (KR-20). The total 33-item scale showed adequate internal consistency (KR-20 = 0.759). Domain-level KR-20 coefficients were 0.462 for general HPV knowledge, 0.481 for HPV screening-test knowledge, 0.416 for HPV vaccine-protection knowledge, and 0.479 for knowledge of the current HPV vaccination program. These domain-level estimates were taken into consideration when interpreting analyses based on the individual knowledge domains. Because continuous variables did not satisfy the assumption of normal distribution, nonparametric methods were used for group comparisons. The Mann–Whitney U test was applied to compare HPV knowledge scores between two groups, including sex, personal HPV vaccination status, recommending the HPV vaccine to patients, and intention to receive the HPV vaccine. Comparisons among three professional groups (non-specialist family physicians, family medicine specialists, and family medicine residents) and parental/child gender status (no children, children without a daughter, and children with at least one daughter) were performed using the Kruskal–Wallis test. When a statistically significant difference was identified, post hoc pairwise comparisons were conducted using the Dunn–Bonferroni test. Binary logistic regression analysis was performed to identify independent determinants of recommending the HPV vaccine to patients. The dependent variable, recommendation of the HPV vaccine to patients, was coded as yes = 1 and no = 0. A hierarchical modeling strategy was applied in two blocks. In the first block, sociodemographic variables and relevant individual characteristics (age retained as a continuous predictor to preserve statistical power, sex, profession, marital status, personal HPV vaccination status, and parental/child gender status categorized as no children, children without a daughter, and children with at least one daughter) were entered into the model. In the second block, HPV knowledge domains (general HPV knowledge, knowledge of HPV screening tests, knowledge of HPV vaccine protection, and knowledge of the current HPV vaccination program) were added. For categorical variables, the simple contrast method was used, with the first category serving as the reference. Results were reported as adjusted odds ratios (aORs = Exp(B)) with 95% confidence intervals and *p*-values. Model fit was assessed using omnibus tests and the Hosmer–Lemeshow goodness-of-fit test, whereas overall model fit and explanatory capacity were evaluated using Cox–Snell and Nagelkerke pseudo-R^2^ metrics.

A two-tailed *p*-value < 0.05 was considered statistically significant for all analyses. Furthermore, no formal adjustment (e.g., Bonferroni correction) was applied for multiple testing across the pairwise Mann–Whitney U comparisons in the pairwise analysis; consequently, these specific *p*-values should be interpreted as exploratory.

### 2.5. Sample Size and Precision

Given the logistical constraints of open-link digital recruitment and national personal data protection regulations (KVKK), which restricted access to individual contact rosters, recruitment was conducted pragmatically through national primary care networks. No formal prospective power calculation was performed. An initial operational target of 163 participants was used as a recruitment benchmark based on feasibility rather than as a formal power-based sample size requirement. Data collection continued until 238 valid and fully completed questionnaires were obtained. For an estimated national population of approximately 35,000 family physicians, the final sample of 238 corresponded to a margin of error of 6.33% at the 95% confidence level. This achieved precision was considered adequate for the descriptive objectives of the study, while the multivariable regression findings were interpreted cautiously in view of the number of outcome events and model parameters.

### 2.6. Ethical Considerations

Prior to the initiation of the study, ethical approval was obtained from the Local Scientific Medical Research Ethics Committee of the Faculty of Medicine, Karamanoğlu Mehmetbey University, Karaman, Türkiye (Approval No. 07-2024/04; 11 June 2024).

Participants were presented with an informed consent statement on the first page of the online questionnaire and provided consent by clicking the “I agree to participate” option. Participation was entirely voluntary, and no personally identifiable information was collected to ensure participant confidentiality and privacy.

## 3. Results

### 3.1. Participant Characteristics and Perspectives on HPV Vaccination

A total of 238 valid and completed questionnaires were received and analyzed from family physicians across Türkiye. Due to the open-link nature of the online distribution through professional primary care networks and instant messaging groups, the exact response rate could not be calculated; however, the final sample size (*n* = 238) safely exceeded the predefined minimum target of 163 participants. Of the participants, 60.1% were female and 39.9% were male. The mean age was 36.29 ± 7.76 years, with a median of 35.0 years (IQR: 30.0–41.3; range: 24–58). Most participants were married (81.1%), while 17.2% were single and 1.7% were widowed or divorced. Overall, 34.5% had no children, whereas 65.5% had at least one child. Approximately half of the participants did not have a daughter (50.4%), while 49.6% had at least one daughter. In addition, 39.1% of the participants had at least one son, whereas 60.9% had no sons. Regarding professional status, 16.0% of the participants were non-specialist family physicians, 42.0% were family medicine specialists, and 42.0% were family medicine residents. Only 15.1% of participants had received the HPV vaccine themselves, whereas 84.9% had not. Among those who had not been vaccinated, 40.6% reported considering HPV vaccination, 41.6% stated that they were not considering it, and 17.8% were undecided. When intention to vaccinate existing or future children against HPV was evaluated, 88.2% of the participants reported that they were considering HPV vaccination for at least one child. Among them, 61.3% indicated that they would vaccinate both daughters and sons, 26.5% only daughters, and 0.4% only sons. In contrast, 11.8% stated that they did not intend to vaccinate their children against HPV.

Overall, 81.1% of participants reported recommending the HPV vaccine to their patients. In addition, 83.6% believed that the HPV vaccine should be included in the national immunization schedule, whereas 5.9% opposed its inclusion and 10.5% were undecided. Detailed participant characteristics and perspectives on HPV vaccination are presented in Table 1.

When analyzed by sex, the rate of HPV vaccine recommendation was significantly higher among female participants (85.3%) than among male participants (74.7%) (Pearson’s χ^2^ = 4.17, df = 1, *p* = 0.041). By contrast, no statistically significant difference in HPV vaccine recommendation was observed between participants younger than 40 years (80.6%) and those aged 40 years or older (82.1%) (χ^2^ = 0.07, df = 1, *p* = 0.792).

Regarding systemic awareness of public health frameworks, baseline familiarity with national guidelines was notably varied among participants. While 83.6% of family physicians demonstrated positive systemic awareness regarding the strategic necessity of incorporating the HPV vaccine into the national immunization schedule (Table 1), awareness of specific operational updates within the national program remained suboptimal, as reflected by the lower domain scores for current program parameters (as detailed below) Furthermore, overall awareness regarding routine national screening infrastructure (KETEM and primary care co-testing protocols) was high, whereas awareness regarding out-of-pocket regulatory pricing status directly influenced clinical recommendation patterns.

### 3.2. Reasons for Not Recommending the HPV Vaccine

Among the 45 participants who reported that they did not recommend the HPV vaccine to their patients, one did not respond to the question regarding the reasons for non-recommendation; therefore, the analysis was based on 44 participants. A total of 61 responses were obtained. The most frequently reported reasons were the high cost of the vaccine (36.4%) and insufficient knowledge to educate patients about the vaccine (36.4%), followed by lack of sufficient time to educate patients about the vaccine (34.1%). Concerns about vaccine safety and side effects were reported less frequently (22.7%). Rarely reported reasons included concern that the vaccine might encourage early or risky sexual behavior among adolescents (6.8%) and the perception that vaccination was unnecessary because it is not included in the national immunization program (2.3%) (Table 2).

### 3.3. HPV Knowledge Scores

Overall, participants demonstrated high levels of HPV-related knowledge. The mean general HPV knowledge score was 13.79 ± 1.46 (median: 14.0; IQR: 13.0–15.0; range: 5–16). The mean score for knowledge of HPV screening tests was 5.04 ± 1.12 (median: 5.0; IQR: 5.0–6.0; range: 1–6). The mean score for knowledge of the protective efficacy of the HPV vaccine was 4.47 ± 0.82 (median: 5.0; IQR: 4.0–5.0; range: 1–5).

By contrast, the mean score for knowledge of the current HPV vaccination program was lower (3.49 ± 1.43; median: 4.0; IQR: 3.0–5.0; range: 0–6) (Table 3).

### 3.4. Comparison of HPV Knowledge Scores

HPV knowledge scores differed significantly according to sex and, more prominently, according to the behavior of recommending the HPV vaccine to patients. Female participants had significantly higher scores than males for knowledge of the protective efficacy of the HPV vaccine (*p* = 0.045) and knowledge of the current HPV vaccination program (*p* < 0.001). Participants who had received the HPV vaccine themselves also had significantly higher scores for knowledge of the current HPV vaccination program than those who had not (*p* = 0.006).

In addition, physicians who recommended the HPV vaccine to their patients had significantly higher scores across all knowledge domains, including general HPV knowledge (*p* = 0.004), knowledge of HPV screening tests (*p* = 0.008), knowledge of HPV vaccine protection (*p* = 0.004), and knowledge of the current HPV vaccination program (*p* = 0.003). By contrast, having children, having a daughter, and individual intention to receive the HPV vaccine were not significantly associated with HPV knowledge levels (*p* > 0.05) (Table 4).

### 3.5. Comparison of HPV Knowledge Scores by Professional Group

Significant differences were observed across professional groups in general HPV knowledge (*p* < 0.001). Pairwise comparisons showed that family medicine specialists had significantly higher general HPV knowledge scores than non-specialist family physicians (*p* = 0.009) and family medicine residents (*p* = 0.002). However, no significant difference was found between non-specialist family physicians and residents (*p* > 0.999).

Similarly, knowledge of HPV screening tests differed significantly among professional groups (*p* < 0.001). Post hoc analyses indicated that family medicine specialists had significantly higher screening test knowledge scores than residents (*p* < 0.001), whereas no significant differences were observed between non-specialist family physicians and specialists (*p* = 0.821) or between non-specialist family physicians and residents (*p* = 0.284).

Knowledge regarding the protective efficacy of the HPV vaccine also differed significantly across professional groups (Kruskal–Wallis, p = 0.007). Pairwise comparisons revealed a significant difference between non-specialist family physicians and family medicine specialists (*p* = 0.013).

A significant difference was also found in knowledge of the current HPV vaccination program among professional groups (*p* = 0.021). Post hoc analyses showed that family medicine specialists had significantly higher scores than non-specialist family physicians (*p* = 0.037), while the differences between non-specialist family physicians and residents (*p* > 0.999) and between specialists and residents (*p* = 0.114) were not statistically significant (Table 5).

### 3.6. Factors Associated with HPV Vaccine Recommendation

Evaluation of the two-step logistic regression models showed that both models had good fit (Model 1: Hosmer–Lemeshow χ^2^ (df = 8) = 4.282, *p* = 0.831; Model 2: Hosmer–Lemeshow χ^2^ (df = 8) = 7.492, *p* = 0.485). Model 1, which included only demographic and individual variables, was not statistically significant (*p* = 0.091) and explained 9% of the variance in HPV vaccine recommendation behavior. By contrast, Model 2 demonstrated a robust and statistically significant block improvement upon the hierarchical addition of HPV knowledge variables (Δχ^2^ = 17.114; *p* = 0.002), with the total explained variance increasing to 19%.

In Model 1, all baseline demographic and professional variables—including age, sex, occupation, marital status, the re-specified three-level child-status variable, and personal HPV vaccination status—were not significantly associated with HPV vaccine recommendation behavior and were interpreted as null findings. The only individual parameter approaching the conventional significance threshold was personal HPV vaccination status (not vaccinated vs vaccinated; OR = 0.245; 95% CI: 0.054–1.105; *p* = 0.067); however, it did not reach statistical significance, and the overall omnibus test of the baseline model was also non-significant (χ^2^(8) = 12.344; *p* = 0.136). Neither family medicine specialist status (OR = 0.851; 95% CI: 0.310–2.331; *p* = 0.753) nor family medicine resident status (OR = 1.004; 95% CI: 0.357–2.827; *p* = 0.994), compared with non-specialist family physicians, was associated with HPV vaccine recommendation behavior.

In the final Model 2, HPV-related knowledge variables were added. Among these, knowledge of the current national HPV vaccination program showed the strongest, although not statistically significant, association with the likelihood of recommending the HPV vaccine to patients (OR = 1.286; 95% CI: 0.984–1.680; *p* = 0.066). No significant associations were observed between the other knowledge domains and recommendation behavior. Nevertheless, the addition of the four knowledge domains significantly improved model fit (Δχ^2^(4) = 17.114; *p* = 0.002), while Nagelkerke’s pseudo-R^2^ increased from 0.081 to 0.188. Similarly, occupation remained non-significant in the adjusted model; compared with non-specialist family physicians, neither family medicine specialists (OR = 0.519; 95% CI: 0.170–1.586; *p* = 0.250) nor family medicine residents (OR = 0.962; 95% CI: 0.312–2.972; *p* = 0.947) differed significantly in HPV vaccine recommendation behavior. Demographic and family-related variables also remained non-significant after the inclusion of HPV knowledge variables (Table 6).

## 4. Discussion

To contextualize our findings, it is essential to consider Türkiye’s unique healthcare framework. While the country provides a robust, fully subsidized screening infrastructure (utilizing HPV-DNA and Pap smear co-testing via Family Health Centers and KETEM), HPV vaccination remains entirely voluntary and out-of-pocket. In a system relying on opportunistic clinical encounters rather than institutional mandates, family physicians’ baseline knowledge and awareness serve as the primary bridge to public vaccine acceptance. Therefore, evaluating these frontline providers is critical for identifying communication barriers and designing targeted public health interventions tailored to this specific primary care context.

In our study, 81.1% of family physicians reported recommending the HPV vaccine to their patients, a rate higher than several previous national studies [26,28,32,33]. This variation across the Turkish literature, where rates range from 41% to 85% among different specialties [26,28,32,33,34,35,36], may be attributable to broader regional representation or a chronological increase in vaccine acceptance over time. In countries where the HPV vaccine is integrated into national immunization programs, near-universal provider recommendation is typically sustained through official institutional endorsements, comprehensive cost coverage, and systemic guidelines that build clinical confidence [22,23,25,29]. Although the HPV vaccine sits outside the routine schedule in Türkiye, the high recommendation rate observed here, coupled with the fact that 83.6% of participants believe it should be nationally scheduled, demonstrates a strong proactive clinical consensus. This consensus is mirrored internationally; for instance, a study among primary care clinicians reported that 85.5% of physicians recommended the HPV vaccine for young women, and 68.3% strongly believed that the vaccine should be fully integrated into the national immunization schedule [37]. The alignment between these data underscores a consistent global demand among primary care providers for state-led immunization frameworks.

Another important finding was the participants’ intention to vaccinate their own children against HPV. Although HPV vaccination has traditionally been framed around females, 61.3% of our participants stated they would vaccinate both daughters and sons. Crucially, 39.1% of the overall study population parented at least one son, illustrating a relevant demographic context for gender-neutral approaches. This recognition aligns with global shifts observed after policy expansions; for instance, following the extension of guidelines to males in France, family physicians demonstrated robust clinical practices toward vaccinating both boys and girls actively [29]. Conversely, in female-only frameworks—such as that evaluated in Hong Kong prior to the introduction of its school-based HPV immunization program (Wong et al., 2013) [37]—recommendation rates for male adolescents remained critically low. Despite local out-of-pocket costs and exclusion from the routine national program in Türkiye, our participants’ strong willingness to immunize both daughters and sons positions them in close alignment with current global gender-neutral vaccination recommendations [22,29]. Interestingly, our findings revealed a divergence from theoretical expectations regarding child gender. While experiential crossover literature posits that clinicians raising daughters show higher vaccine advocacy, our initial baseline model indicated an unexpected borderline inverse trend for clinicians with daughters alongside a trend toward lower current program knowledge. Methodologically, this parameter was subject to sample distribution strain prior to model re-specification, reflecting statistical instability rather than a true negative behavioral barrier. Contextually, clinician-parents in Türkiye facing fully out-of-pocket vaccine costs for their own families may experience distinct economic dilemmas compared to routine clinical counseling. While this estimate remains too unstable to support definitive causal claims, directly acknowledging this divergence highlights the complex interplay between personal parental context and clinical recommendation in non-subsidized primary care settings.

Nevertheless, vaccine cost and a lack of specific program information emerged as the most prominent barriers to vaccine recommendation, echoing previous national findings [26,28]. Within the Turkish primary care context, the exclusion of the vaccine from the national schedule shifts the financial burden onto individuals, making it more difficult for the vaccine to become established as a routine preventive service. This out-of-pocket financial barrier underscores a universal public health challenge. International evidence consistently confirms that removing financial constraints is a fundamental prerequisite for successful immunization. For instance, structured public programs in the United States, such as the federally funded Vaccines for Children (VFC) program [38] and state-level policy efforts in Oregon [39], successfully maximized HPV vaccine uptake by completely eliminating financial barriers for eligible adolescents. These global precedents indicate that high baseline awareness among clinicians is most effective when structurally accompanied by cost-mitigation strategies.

When placed against the broader international literature, these local structural and knowledge-based dynamics both support established global paradigms and add distinct new evidence to our understanding of provider behaviors. On one hand, our emphasis on the critical role of family physicians strongly supports the previously noted systematic review confirming that direct provider recommendation is a vital factor in initiating the vaccination series [24]. Furthermore, our conclusion that addressing information gaps is vital agrees with global evidence showing that provider-directed interventions, such as targeted education and monitoring, consistently enhance vaccine utilization [40]. On the other hand, regarding the specific barriers preventing recommendation, our data refines and contrasts with some global trends. While multi-country systematic reviews and multi-specialty studies frequently cite doubts about vaccine safety and necessity [23], concerns over side effects [25], or diminishing trust in health authorities [41] as primary impediments, our findings demonstrate that Turkish primary care providers face obstacles rooted heavily in out-of-pocket costs and policy integration gaps rather than clinical skepticism. Additionally, whereas some high-recommendation cohorts in the United States did not explore the reasons for non-recommendation in depth [42], our results highlight how structural limitations intersect with specific knowledge domains. Taken together, these insights demonstrate that continuous professional education alone is insufficient to overcome the practical limitations imposed by current policy gaps, and localized training must be paired with broader system-level pathways to optimize real-world vaccine accessibility.

To address the identified barriers, a multi-level framework can be utilized. At the policy level, out-of-pocket costs could be mitigated through targeted vaccine subsidies or co-payment models, alongside future evaluation for national schedule integration. At the facility level, structural time constraints within busy primary care practices can be optimized through task-sharing, where trained public health nurses assist in patient education and vaccine screening. Finally, at the provider level, localized policy knowledge gaps can be systematically addressed through Continuous Medical Education (CME) modules and digital decision-support tools within existing electronic health record systems. Aligning these strategies offers a pragmatic approach to enhance preventive care delivery in primary care settings.

Regarding gender-based dynamics, female participants demonstrated higher knowledge scores and recommended the vaccine significantly more frequently than their male counterparts. While some mixed-cohort studies including both physicians and nurses have reported alternative trends [27,43], our findings align with research conducted exclusively among Turkish family physicians showing higher female knowledge [28] and recommendation rates [26]. This is also consistent with a global systematic review confirming that female healthcare professionals exhibit a higher likelihood of vaccine recommendation [23]. Furthermore, the higher recommendation rates observed among female clinicians are primarily driven by routine clinical practice patterns. More frequent communication with patients regarding reproductive health and cervical screening provides female physicians with greater practical familiarity and enhanced clinical counseling capacity regarding HPV vaccination. It should be noted, however, that while the association between physician sex and recommendation rate was statistically significant in bivariate analysis (*p* = 0.041), this difference lost statistical significance after multivariable adjustment (*p* = 0.088). This attenuation further suggests that routine clinical exposure and practice patterns exert a more decisive influence than physician sex per se.

Consistent with this behavioral link, physicians who recommended the HPV vaccine possessed significantly higher scores across all knowledge domains, indicating an association between comprehensive HPV-related knowledge and clinical recommendation patterns. In the hierarchical logistic regression analysis, the baseline model containing demographic, professional, and individual variables was not statistically significant (χ^2^(8) = 12.344, *p* = 0.136; Nagelkerke pseudo-R^2^ = 0.081). However, the addition of the four HPV knowledge domains significantly improved model fit (Δχ^2^(4) = 17.114, *p* = 0.002), and the final model was statistically significant (χ^2^(12) = 29.459, *p* = 0.003; Nagelkerke pseudo-R^2^ = 0.188). This improvement suggests that the knowledge domains contribute collectively to the model’s explanatory capacity rather than operating as isolated predictors. Furthermore, because individual sub-scales did not retain independent statistical significance in the fully adjusted model, clinician knowledge is best understood as an integrated framework that operates alongside, and is conditioned by, broader structural and financial realities.

It is also noteworthy that 15.1% of participants had received the HPV vaccine themselves and 40.6% of the unvaccinated subgroup were considering uptake, rates higher than those reported in older national cohorts [28,32]. To contextualize this personal uptake, the cohort’s age profile (median: 35.0 years, Q3: 41.3 years) suggests that the majority of our sample was historically within the standard age-eligibility windows when vaccines were licensed in Türkiye in 2007. While the senior cohort faced strict historical product indications that limited early access, our multivariable model demonstrated that participant age was not independently associated with clinical HPV vaccine recommendation behavior (OR = 1.015, 95% CI: 0.955–1.079; *p* = 0.639). This underscores that while historical timelines unevenly shaped the physicians’ personal immunization histories, their role as clinical advocates remains uniformly strong across all age groups. However, given the cross-sectional nature of this study, these age-eligibility interactions should be interpreted cautiously as descriptive conjectures rather than definitive causal inferences. These statistical findings indicate that individual clinician knowledge does not operate as an isolated predictor, but is instead significantly influenced by broader structural and financial barriers. Indeed, the fact that personal vaccination rates remain limited suggests that structural barriers continue to influence individual choices, which means that accessibility and cost must be reduced to fully translate positive provider attitudes into personal vaccination behavior.

This conceptual divergence in the multivariable analysis can be explained by both methodological and contextual limitations. Methodologically, a potential ceiling effect and subsequent restricted variance are common when surveying highly educated healthcare professionals, which mathematically constrains the model’s capacity to isolate independent statistical associations. Contextually, the final multivariable model yielded a Nagelkerke pseudo-R^2^ of 0.188, indicating improved but still limited explanatory capacity for HPV vaccine recommendation behavior. This finding suggests that the measured cognitive domains contribute partially to recommendation behavior but do not constitute a comprehensive explanatory framework. Proactive immunization counseling in primary care is a complex behavior shaped by unmeasured institutional and environmental variables. Systemic factors such as institutional policies, daily clinical workloads, patient-driven demand, and immediate local reimbursement mechanisms likely play substantial roles in shaping a physician’s routine counseling capacity, suggesting that optimizing public health outcomes involves aligning educational initiatives with supportive workplace infrastructures.

Notably, participants demonstrated lower knowledge scores regarding the current national program parameters compared to clinical or screening domains. This disparity suggests that because the vaccine sits outside Türkiye’s routine immunization schedule, policy-specific details remain less visible within standard medical education and daily primary care practice. Consequently, future educational initiatives for family physicians should expand beyond traditional epidemiological features to actively incorporate country-specific vaccination frameworks and implementation policies.

Regarding age dynamics, no statistically significant association was found with overall HPV knowledge levels, contrasting with some national data showing that knowledge increases with age [26]. This lack of age-dependent variance may be explained by the recent, up-to-date training received by younger physicians and residents during medical school, a perspective supported by global data indicating that recent graduates often maintain a stronger focus on preventive medicine [23]. Additionally, the relatively narrow age distribution within our sample may have limited the detection of broader age-related differences.

Finally, differences across professional groups were selective rather than uniform across all knowledge domains. Family medicine specialists had higher general HPV knowledge scores than both non-specialist family physicians (*p* = 0.009) and family medicine residents (*p* = 0.002). For HPV screening-test knowledge, specialists scored higher than residents (*p* < 0.001), but did not differ significantly from non-specialist family physicians (*p* = 0.821). For knowledge of HPV vaccine protection, specialists differed significantly only from non-specialist family physicians (*p* = 0.013), whereas the difference from residents was not statistically significant (*p* = 0.070). Similarly, specialists had higher knowledge of the current HPV vaccination program than non-specialist family physicians (*p* = 0.037), but not than residents (*p* = 0.114). The remaining pairwise comparisons were not statistically significant. Furthermore, vaccine recommendation rates were not directly compared across professional groups, and occupation was not independently associated with HPV vaccine recommendation in either hierarchical regression model (Model 1 overall *p* = 0.897; Model 2 overall *p* = 0.289). These findings suggest that postgraduate specialty training may be associated with advantages in specific HPV-related knowledge domains; however, they do not support a consistent difference across all domains or in vaccine recommendation behavior. Accordingly, continuing medical education should focus on specific knowledge gaps across professional groups rather than being directed exclusively according to professional status.

### Strengths and Limitations

One of the main strengths of this study is that it is among the limited number of studies in Türkiye that have evaluated family physicians’ knowledge levels and awareness regarding HPV infection and HPV vaccination together. The inclusion of participants from different regions of Türkiye contributed to a broader interpretation of the findings. In addition, the study addressed the topic from a multidimensional perspective by evaluating not only knowledge levels, but also HPV vaccine recommendation behavior, personal vaccination status, and opinions regarding the national immunization program. The use of an HPV knowledge scale with established validity and reliability further strengthened the methodological rigor of the study.

However, several limitations should also be considered. First, because of the cross-sectional design, the relationships between variables cannot be interpreted as causal. Second, since the data were collected through self-report, social desirability bias and recall bias may have affected the findings. The relatively young mean age of the participants may also have influenced both knowledge levels and the tendency to recommend or receive the HPV vaccine, as younger physicians may have had more up-to-date knowledge regarding HPV infection and vaccination. In addition, the online data collection method may have introduced selection bias by increasing the participation of physicians who use digital tools more actively or who are more interested in the topic. Therefore, these limitations should be taken into account when interpreting the findings. Additionally, a key statistical limitation pertains to the events-per-variable (EPV) ratio in our multivariable logistic regression models. With 45 non-recommending physicians defining the minor outcome category, Model 1 operated at approximately 5.6 EPV, whereas the fully adjusted Model 2—which incorporated twelve degrees of freedom—fell below 4 EPV. Because Model 2 serves as the primary framework foregrounded throughout our Discussion and Conclusions, the individual predictor estimates within the adjusted model should be interpreted with appropriate caution due to inherent sample distribution strain and potential parameter instability.

## 5. Conclusions

This study demonstrates that family physicians in Türkiye possess high baseline knowledge regarding HPV infection and vaccination, with a majority actively recommending it in primary care. Crucially, these dynamics demonstrate that primary care immunization advocacy is shaped by the combined influence of professional knowledge and overriding structural factors. While unadjusted analyses indicate a positive link between higher knowledge scores and clinical advocacy, multivariable adjustment reveals that individual knowledge domains do not act as independent standalone predictors, with only knowledge of the current national program updates showing a borderline trend. Nevertheless, the collective contribution of the knowledge block to the overall model fit underscores that sustaining high professional knowledge remains most effective when aligned with supportive public health initiatives. Although personal vaccination status was not significantly associated with recommendation behavior in our study (OR = 0.34, *p* = 0.169), expanding public health policy frameworks to facilitate the vaccination of primary care providers themselves remains a valuable broader policy recommendation. Supporting the immunization of front-line clinicians can safeguard their personal health and foster overall institutional vaccine advocacy in the community. Ultimately, integrating provider-focused support with existing primary care screening frameworks will contribute significantly to national cervical cancer prevention goals.

## Figures and Tables

**Table 1:** Participants’ sociodemographic characteristics and perspectives on the HPV vaccine (*n* = 238).

Variable	Category	*n*	%
Age	Mean in years	238	36.29 (SD ± 7.76)
	Median in years (Q1–Q3)	238	35.0 (30.0–41.3)
	Range in years	238	24–58
Gender	Female	143	60.1
	Male	95	39.9
Marital status	Single	41	17.2
	Married	193	81.1
	Widowed/Divorced	4	1.7
Total number of children	0	82	34.5
	1	61	25.6
	2	65	27.3
	≥3	30	12.6
Child status	No children	82	34.5
	Children without a daughter	38	16.0
	At least one daughter	118	49.5
Number of daughters	0	120	50.4
	1	81	34.0
	≥2	37	15.5
Number of sons	0	145	60.9
	1	66	27.7
	≥2	27	11.3
Occupation	Non-specialist Family physician	38	16.0
	Family medicine specialist	100	42.0
	Family medicine resident (AHU/SAHU)	100	42.0
Have you had the HPV vaccine?	Yes	36	15.1
	No	202	84.9
Considering getting the HPV vaccine (*those who haven’t been vaccinated*)	Yes	82	40.6
	No	84	41.6
	Undecided	36	17.8
Intention to have your child vaccinated against HPV	No	28	11.8
	Only to a girl	63	26.5
	Only to a boy	1	0.4
	To both a girl and a boy	146	61.3
Recommending the HPV vaccine to patients	Yes	193	81.1
	No	45	18.9
Should the HPV vaccine be included in the national immunization program?	Yes	199	83.6
	No	14	5.9
	Undecided	25	10.5

**Table 2:** Reasons reported by participants for not recommending the HPV vaccine to their patients (*n* = 44; multiple responses allowed).

Reason	*n*	Responses (%)	Participants Selecting This Reason (%) *
The vaccine is expensive	16	26.2	36.4
Lack of sufficient knowledge to educate patients about the vaccine	16	26.2	36.4
Lack of sufficient time to educate patients about the vaccine	15	24.6	34.1
Concerns about the vaccine’s reliability/side effects	10	16.4	22.7
Concern that the vaccine may increase early/risky sexual behavior in adolescents	3	4.9	6.8
Not considering it necessary because it is not part of the mandatory vaccination schedule	1	1.6	2.3
Total	61	100.0	-

* Footnote: Multiple responses were allowed; therefore, the percentages in the last column do not sum to 100%.

**Table 3:** Descriptive statistics of HPV knowledge scores (*n* = 238).

Variable	Mean ± SD	Median (Q1–Q3)	Min–Max
General HPV knowledge (0–16)	13.79 ± 1.46	14.0 (13.0–15.0)	5–16
HPV screening test knowledge (0–6)	5.04 ± 1.12	5.0 (5.0–6.0)	1–6
HPV vaccine knowledge—protection (0–5)	4.47 ± 0.82	5.0 (4.0–5.0)	1–5
Current HPV vaccination program knowledge (0–6)	3.49 ± 1.43	4.0 (3.0–5.0)	0–6

**Table 4:** Comparison of HPV knowledge scores according to sociodemographic characteristics and vaccination attitudes.

		General HPV Information		HPV Screening Test Information		HPV Vaccine Information (Protection)		HPV Vaccination Program Information	
		Median (Q1–Q3)	*p* *	Median (Q1–Q3)	*p* *	Median (Q1–Q3)	*p* *	Median (Q1–Q3)	*p* *
Gender	Female (*n* = 143)	14 (13–15)	0.718	5 (5–6)	0.304	5 (4–5)	0.045	4 (3–5)	<0.001
	Male (*n* = 95)	14 (13–15)		5 (4–6)		5 (4–5)		3 (2–4)	
Having children	No (*n* = 82)	14 (13–15)	0.819	5 (4–6)	0.307	5 (4–5)	0.850	4 (3–5)	0.316
	Yes (*n* = 156)	14 (13–15)		5 (5–6)		5 (4–5)		4 (3–5)	
Having a daughter	No (*n* = 120)	14 (13–15)	0.906	5 (5–6)	0.354	5 (4–5)	0.364	4 (3–5)	0.075
	Yes (*n* = 118)	14 (13–15)		5 (4–6)		5 (4–5)		4 (2–4)	
Getting the HPV vaccine for themselves	Yes (*n* = 36)	14 (14–15)	0.118	6 (5–6)	0.170	5 (4–5)	0.127	4 (3–5)	0.006
	No (*n* = 202)	14 (13–15)		5 (4–6)		5 (4–5)		4 (2–4)	
Intention to get the HPV vaccine (among those not vaccinated) **	Yes (*n* = 82)	14 (13–15)	0.572	5 (5–6)	0.092	5 (4–5)	0.504	4 (3–5)	0.169
	No (*n* = 84)	14 (13–15)		6 (5–6)		5 (4–5)		4 (2–4)	
Recommending the HPV vaccine to patients	Yes (*n* = 193)	14 (13–15)	0.004	5 (5–6)	0.008	5 (4–5)	0.004	4 (3–5)	0.003
	No (*n* = 45)	13 (12–14)		5 (3.5–6)		4 (3–5)		3 (1–4)	

* Mann–Whitney U. Note: No correction for multiple testing was applied to the pairwise comparisons.** Comparison of HPV knowledge scores by intention to receive the HPV vaccine was conducted between participants with affirmative intention (*n* = 82) and those without intention (*n* = 84); undecided participants (*n* = 36) were excluded from this specific binary comparison.

**Table 5:** Comparison of HPV knowledge scores by professional groups.

		*n*	Median	Q1	Q3	*p* *	*p*1	*p*2	*p*3
General HPV knowledge	Non-specialist Family physician (1)	38	14.0	12.0	15.0	<0.001	0.009	>0.999	0.002
	Family medicine specialist (2)	100	14.0	14.0	15.0				
	Family Medicine resident (3)	100	14.0	13.0	14.8				
HPV screening test knowledge	Non-specialist Family physician (1)	38	5.0	5.0	6.0	<0.001	0.821	0.284	<0.001
	Family medicine specialist (2)	100	6.0	5.0	6.0				
	Family Medicine resident (3)	100	5.0	4.0	6.0				
General HPV vaccine information on protection	Non-specialist Family physician (1)	38	4.5	3.8	5.0	0.007	0.013	0.736	0.070
	Family medicine specialist (2)	100	5.0	4.0	5.0				
	Family Medicine resident (3)	100	5.0	4.0	5.0				
Knowledge of the current HPV vaccination program	Non-specialist Family physician (1)	38	3.0	2.0	4.0	0.021	0.037	>0.999	0.114
	Family medicine specialist (2)	100	4.0	3.0	5.0				
	Family Medicine resident (3)	100	4.0	2.0	4.0				

*p* *: Kruskal–Wallis test (three groups). *p*1: 1 vs. 2; *p*2: 1 vs. 3; *p3*: 2 vs. 3 (pairwise post hoc comparisons).

**Table 6:** Effects of demographic and individual factors and HPV knowledge levels on HPV vaccine recommendation behavior.

Variable	B	S.E.	Wald	*p* *	OR	OR (95% CI)
**Model 1**						
Age	0.021	0.03	0.50	0.478	1.02	0.964–1.080
Gender (Male vs. Female)	−0.624	0.36	3.00	0.083	0.54	0.265–1.085
Marital status (Married vs. Not married)	−0.222	0.55	0.16	0.688	0.8	0.271–2.363
Occupation (overall)	—	—	0.22	0.897	—	—
Family medicine specialist vs. non-specialist family physician	−0.162	0.51	0.10	0.753	0.85	0.310–2.331
Family medicine resident vs. non-specialist family physician	0.004	0.53	<0.001	0.994	1	0.357–2.827
Personal HPV vaccination status (Not vaccinated vs. Vaccinated)	−1.406	0.77	3.35	0.067	0.25	0.054–1.105
Child status (overall)	—	—	2.75	0.252	—	—
Children without a daughter vs. No children	0.924	0.68	1.85	0.174	2.52	0.666–9.531
At least one daughter vs. No children	−0.037	0.51	0.01	0.942	0.96	0.352–2.637
**Model 2**						
Age	0.015	0.03	0.22	0.639	1.02	0.955–1.079
Gender (Male vs. Female)	−0.440	0.39	1.29	0.256	0.64	0.301–1.377
Marital status (Married vs. Not married)	−0.446	0.59	0.57	0.45	0.64	0.201–2.038
Occupation (overall)	—	—	2.49	0.289	—	—
Family medicine specialist vs. non-specialist family physician	−0.656	0.57	1.32	0.25	0.52	0.170–1.586
Family medicine resident vs non-specialist family physician	−0.038	0.58	0.00	0.947	0.96	0.312–2.972
Personal HPV vaccination status (Not vaccinated vs. Vaccinated)	−1.077	0.78	1.91	0.167	0.34	0.074–1.567
Child status (overall)	—	—	1.72	0.424	—	—
Children without a daughter vs. No children	0.871	0.71	1.51	0.218	2.39	0.597–9.558
At least one daughter vs. No children	0.165	0.55	0.09	0.763	1.18	0.403–3.448
General knowledge about HPV	0.161	0.13	1.46	0.227	1.17	0.905–1.523
Knowledge about HPV screening tests	0.239	0.17	2.10	0.148	1.27	0.919–1.753
Knowledge about HPV vaccine protection	0.169	0.24	0.51	0.475	1.18	0.745–1.880
Knowledge about the current HPV vaccination program	0.251	0.14	3.39	0.066	1.29	0.984–1.680

* Binary logistic regression. OR: odds ratio; CI: confidence interval. Reference categories were female, not married, non-specialist family physician, personally vaccinated, and no children. Because only four participants were widowed or divorced, marital status was dichotomized as married versus not married; the not-married category included single, widowed, and divorced participants. Model 1 included sociodemographic, professional, and individual variables. Model 2 additionally included HPV knowledge variables. Model fit statistics were as follows: Model 1, omnibus *p* = 0.136, Hosmer–Lemeshow *p* = 0.704, Nagelkerke R^2^ = 0.081, −2 log likelihood = 218.459; Model 2, omnibus *p* = 0.003, Hosmer–Lemeshow *p* = 0.428, Nagelkerke R^2^ = 0.188, −2 log likelihood = 201.344. The addition of the HPV knowledge variables significantly improved model fit (Δχ^2^ = 17.114, df = 4, *p* = 0.002).

## Data Availability

The data presented in this study are available on request from the corresponding author due to privacy and ethical restrictions.

## Supplementary Materials

The following supporting information can be downloaded at https://www.mdpi.com/article/10.3390/healthcare14162457/s1, File S1: The STROBE reporting checklist. Refs. [44,45] are cited in Supplementary Materials.
